# Evaluating and Enhancing the Fitness-for-Purpose of Electronic Health Record Data: Qualitative Study on Current Practices and Pathway to an Automated Approach Within the Medical Informatics for Research and Care in University Medicine Consortium

**DOI:** 10.2196/57153

**Published:** 2024-08-19

**Authors:** Gaetan Kamdje Wabo, Preetha Moorthy, Fabian Siegel, Susanne A Seuchter, Thomas Ganslandt

**Affiliations:** 1 Center for Preventive Medicine and Digital Health Baden-Wuerttemberg Department of Biomedical Informatics Medical Faculty of Mannheim, University of Heidelberg Mannheim Germany; 2 Department of Urology and Urosurgery, University Medical Center of Mannheim Medical Faculty of Mannheim University of Heidelberg Mannheim Germany; 3 Medical Center for Information and Communication Technology Erlangen University Hospital Erlangen Germany; 4 Friedrich-Alexander-Universität Erlangen-Nürnberg Erlangen Germany

**Keywords:** data quality, fitness-for-purpose, secondary use, thematic analysis, EHR data, electronic health record, data integration center, Medical Informatics Initiative, MIRACUM consortium, Medical Informatics for Research and Care in University Medicine, data science, integration, data use, visualization, visualizations, record, records, EHR, EHRs, survey, surveys, medical informatics

## Abstract

**Background:**

Leveraging electronic health record (EHR) data for clinical or research purposes heavily depends on data fitness. However, there is a lack of standardized frameworks to evaluate EHR data suitability, leading to inconsistent quality in data use projects (DUPs). This research focuses on the Medical Informatics for Research and Care in University Medicine (MIRACUM) Data Integration Centers (DICs) and examines empirical practices on assessing and automating the fitness-for-purpose of clinical data in German DIC settings.

**Objective:**

The study aims (1) to capture and discuss how MIRACUM DICs evaluate and enhance the fitness-for-purpose of observational health care data and examine the alignment with existing recommendations and (2) to identify the requirements for designing and implementing a computer-assisted solution to evaluate EHR data fitness within MIRACUM DICs.

**Methods:**

A qualitative approach was followed using an open-ended survey across DICs of 10 German university hospitals affiliated with MIRACUM. Data were analyzed using thematic analysis following an inductive qualitative method.

**Results:**

All 10 MIRACUM DICs participated, with 17 participants revealing various approaches to assessing data fitness, including the 4-eyes principle and data consistency checks such as cross-system data value comparison. Common practices included a DUP-related feedback loop on data fitness and using self-designed dashboards for monitoring. Most experts had a computer science background and a master’s degree, suggesting strong technological proficiency but potentially lacking clinical or statistical expertise. Nine key requirements for a computer-assisted solution were identified, including flexibility, understandability, extendibility, and practicability. Participants used heterogeneous data repositories for evaluating data quality criteria and practical strategies to communicate with research and clinical teams.

**Conclusions:**

The study identifies gaps between current practices in MIRACUM DICs and existing recommendations, offering insights into the complexities of assessing and reporting clinical data fitness. Additionally, a tripartite modular framework for fitness-for-purpose assessment was introduced to streamline the forthcoming implementation. It provides valuable input for developing and integrating an automated solution across multiple locations. This may include statistical comparisons to advanced machine learning algorithms for operationalizing frameworks such as the 3×3 data quality assessment framework. These findings provide foundational evidence for future design and implementation studies to enhance data quality assessments for specific DUPs in observational health care settings.

## Introduction

### Insight Into Medical Informatics in Research and Care in University Medicine Data Integration Centers and Data Use Projects

The German Medical Informatics Initiative (MI-I) [1] was launched by the German Federal Ministry of Education and Research to enhance digital health and clinical research infrastructure advancements in Germany. This initiative comprises multiple large consortia. The Medical Informatics in Research and Care in University Medicine (MIRACUM) consortium [2] is among the MI-I consortia that focus on integrating clinical and research data to enhance patient care and facilitate data-driven medical research at German university hospitals. However, MIRACUM includes 10 university hospitals and further medical research institutions across Germany, all of which instantiate medical Data Integration Centers (DICs). The DICs are crucial in gathering, harmonizing, and integrating clinical data from various source systems, including electronic health records (EHRs), clinical imaging systems, and other health-related databases. Additionally, the DICs’ efficient data pipelines support uniform and secure data storage, enabling significant privacy-preserved sharing and analysis of patient data.

Among others, a cornerstone of the MIRACUM consortium’s mission is also to foster data-driven medical research and the improvement of clinical patient care through the implementation of data use projects (DUPs). The DUPs use integrated and harmonized clinical data to answer pertinent research questions, such as identifying patterns, creating predictive models, or supporting evidence-based decision-making in the clinical field. Some of the key investigation areas of focus in the MIRACUM DUPs’ applications so far include personalized medicine [3-5], clinical decision support [6-10], disease monitoring and surveillance [11], drug safety and pharmacovigilance [12], population health management [11], and translational research [13]. Ensuring an appropriate level of data quality (DQ) is imperative for the successful execution of DUPs that use MIRACUM DICs’ clinical data, particularly during the development and implementation of data extraction-transformation-loading [14] processes.

### Evaluating the DQ Impact on Secondary Use: Emphasis on Fitness-for-Purpose

Despite the establishment of robust data integration pipelines within each of the MIRACUM DICs [2,15], improving the fitness-for-purpose of generated data requires overcoming the challenges related to DQ for specific DUP purposes. As a crucial determinant for generating credible evidence [16], high-quality data are essential for drawing sound conclusions. This ensures that a larger target group, for instance, clinicians, health care providers, or givers, may rely on research findings. Conversely, compromised DQ [17] can lead to erroneous results, harmful treatment decisions, or a loss of public trust in the scientific community. However, several studies [18-22] have presented and discussed frameworks and methodologies about how to assess and ensure the quality of EHR data. It remains crucial to gauge the fitness of clinical data to enable achieving designated medical DUP objectives. In the context of observational DIC data-driven studies, for instance, ensuring DQ is of high importance to ensure the validity of the study results, which can emerge as a paramount challenge to overcome. Weiskopf and Weng [20] and Kahn et al [22] emphasized the relevance of adhering to the recommended references, such as the 3×3 data quality assessment (DQA) framework [21], when using EHR data to pursue specific research inquiries. Nevertheless, these current approaches might not comprehensively address the subjective understanding of data fitness that may arise from the diverse backgrounds of secondary data users.

The definition of fitness-for-purpose of clinical data might emphasize greater complexity depending on the requirements of the intended data use or research question to be investigated. In certain DUP contexts, clinical data may be considered as “fit for purpose,” when all eligible patients simply present complete information about specific treatments. In other DUP scenarios, a plausible correlation between these treatments and certain specific diagnostic indicators, laboratory outcomes, or caregiver-related metadata may be additionally required. Girman et al [23] proposed a definition for EHR data fitness-for-purpose, which can be summarized in two dimensions: (1) relevance and (2) reliability. The first dimension, relevance, ensures that the target data elements are available, and a sufficient number of representative patients are present for the study. The second dimension, reliability, verifies whether the data to be used are sufficiently accurate, complete, and traceable (provenance).

However, the interpretation of what constitutes “fitness,” and how to assess it, can substantially vary across different professional domains. For example, a computer scientist might prioritize the algorithmic reliability of data, whereas health care providers might pay attention to the clinical relevance of the data to patient care. These differing perspectives can profoundly affect the ways of assessing data fitness, highlighting the need for more empirical investigation in this domain, especially in multidisciplinary settings such as those of the MIRACUM DICs.

### Prior Works, Contemporary Overview of DQ, and Existing Research Gaps

Against the backdrop described in the previous section, a dynamic DQA tool was designed and implemented [14,24], during the earlier stages of the MIRACUM project, based on the DQA framework suggested by Kahn et al [22]. This tool allows for capturing and assessing DQA metrics, including data completeness, conformance, and plausibility checks, between data sources and target systems to validate DICs’ extraction-transformation-loading processes. Additionally, it facilitates cross-location comparisons of DQ distributions. Furthermore, conducting a DQA study that included comorbidity analysis [25] enriched our initial understanding of strategies to assess the DQ for specific research purposes. However, these significant efforts have yet to comprehensively provide a workable solution for automated assistance for assessing the fitness of DIC data to complete the ongoing or upcoming DUPs.

To bridge the divide between current practices and required improvements, it is imperative to initially capture and understand the activities applied by the MIRACUM DICs to assess and report about the data fitness-for-purpose throughout the data delivery process. Therefore, Reynolds et al [26] introduced objective considerations for evaluating the data fitness-for-purpose, but the authors did not delve deeper into how the DQ checks could be performed based on the research question criteria set by the researchers in an automated way. In contrast, Cho et al [27] developed a fitness-for-purpose tool that assesses data completeness, which may be more practical than intrinsic DQA tools. The predictive data completeness, as proposed by Weiskopf et al [21], which aims to assess, for instance, the impact of missing documentation of data elements such as chronic diseases on the prediction of clinical events, was scarcely addressed. Furthermore, Raman et al [28] suggested conducting study-specific fitness-for-purpose assessment in the early stage of trials and sharing the results in a transparent manner. Nevertheless, knowing whether and how these assessments correlate with successful trials could be valuable. Despite these insights, there is a considerable benefit in exploring common practices and prerequisites for creating a universally deployable solution, spanning multiple sites and systems, and automating the assessment of data fitness-for-purpose.

This research addresses several benefits. On the one hand, it is the first qualitative empirical investigation, gathering and analyzing the experiences in the assessment of data fitness-for-purpose in German medical DICs. This offers the opportunity to gain valuable information about possible existing solutions, challenges, and observable deficits, in comparison with internationally established evidence [21,27,28]. On the other hand, this allows for the investigation of relevant practice-oriented requirements for the development of an evidence-based tool that would enable an automated holistic assessment for DIC data fitness-for-purpose.

### Research Aims

In light of the aforementioned research landscape, this investigation seeks to identify and describe (1) to what extent do the MIRACUM DICs address the fitness-for-purpose of observational health care data and how do the applied approaches contrast with existing recommendations and (2) what key requirements are necessary to develop an automated system within the MIRACUM DICs for assessing the fitness-for-purpose of EHR data for research or clinical applications.

## Methods

### Approach Overview

In this section, we detail the methods used to address the research questions described previously. This encompasses outlining the ethical considerations, the study design, the study sample, and the procedures used to gather and qualitatively analyze the data.

### Ethical Considerations

The study did not require formal ethics approval from the Ethics Committee II of the Medical Faculty Mannheim at the University of Heidelberg, as it involved anonymized data collection that complies with the requirements of the professional code for physicians and the General Data Protection Regulation. Informed consent was obtained from all participants for the participation in the survey on assessing the quality of observational data for secondary use. All data were deidentified to ensure privacy and confidentiality, with no personal information collected or published, and participants received no compensation.

### Study Design

In the course of this investigation, we performed a qualitative study. Therefore, we meticulously implemented a survey across all 10 medical DICs participating in the MIRACUM consortium. Adhering to the directives outlined in the GESIS (Society of Social Science Infrastructure Institutions) survey guideline version 2.0 [29], we developed a survey instrument consisting of 6 open-ended questions (refer to Textbox 1). Such a format facilitates respondents’ ability to articulate their perspectives unrestrictedly, thereby fostering the acquisition of valuable insights and preventing the proliferation of superfluous response alternatives [29]. The instrument was formatted into a questionnaire that was distributed to all participating DIC locations. An accompanying guide was additionally provided during the survey dissemination, which outlined the expected time for the survey completion, the preference for keyword-oriented responses, the intended deadline for response submission, and background information regarding the survey (Multimedia Appendix 1).

**Overview of the survey questionnaire.**

**Metadata related to the location and survey**

- Location name
- Data Integration Center (DIC) data quality (DQ) officer
- Deadline
- Survey feedback completion date

**Metadata related to the survey respondents**

- Gender
- Educational background
- Highest degree
- Years of experience with data quality assessment (DQA)
- Years of experience with observational data fitness-for-purpose assessment

**Survey questions**

- Question 1: On average, how many data requests or local data use projects are handled at your site DIC per quarter?
- Question 2: Which contents are mostly in focus in your local data use projects (eg, care evaluation in transfusion medicine), and which data repositories are most frequently queried in this context (Informatics for Integrating Biology and Bedside, Observational Medical Outcomes Partnership, Fast Healthcare Interoperability Resources, etc)?
- Question 3: How are data use project–specific DQ requirements collected from the perspective of data requesters at their DIC?
- Question 4: In addition to the current Medical Informatics for Research and Care in University Medicine DQA tool, what tools or technical approaches do you use for data use project–specific DQA?
- Question 5: What measures are taken at your location to communicate with data requesting sites about the quality of provided data for the intended purpose, so that data requesters have opportunities to estimate the fitness of the data to complete the intended project?
- Question 6: What would be their expectations or requirements for a fitness-for-purpose cross-site DQ framework that you could adopt in the future to measure DQ related to their data use projects?

### Sample

All medical DICs from the 10 university hospitals affiliated with the MIRACUM consortium were invited to participate in the survey. For each DIC (MIRACUM site), the responsibility of completing the survey was delegated to potential participants. The participants were specialized professionals with expertise in DQ, responsible for evaluating and enhancing the quality of observational data for secondary use within their respective DIC in the context of intern- or cross-location DUPs. While each DIC submitted a single survey response detailing their practice related to assessing data’s fitness-for-purpose, they had the flexibility to involve 1 or multiple participants based on their availability during the data collection period.

### Data Collection

We conducted the data collection from April 15 to June 15, 2022, using a survey questionnaire comprised of 6 open-ended questions. To streamline the survey process for all participating sites and to maintain clear documentation of the participant’s responses, we used Atlassian Confluence (version 7.13.11) [30] as our documentation software.

The data collection process was initiated by extending formal invitations to the DICs through email. These invitations included a link directing to a confluence main page, which outlined the objectives of the survey and provided instructions on how to respond to the survey questions. Additionally, each participating location had access to a distinct embedded confluence-landing subpage, structured with 2 information entry areas. The first input area enabled the entry of meta-information, collecting demographic data on each participant involved in the survey. These included gender, educational background, highest degree obtained, and years of experience in DQA and fitness-for-purpose evaluation. The second area was devoted to gathering specific responses to the 6 open-ended survey questions. Upon completing the meta-information provision and responding to the survey questions, the involved participants completed the survey by submitting their site response and documenting the date of completion.

The survey specifically inquired about the quarterly frequency and current objectives driving observational DUPs within the DICs. Our inquiry was guided by the 3×3 EHR DQA guideline by Weiskopf et al [21], which illustrates the project-specific nature of data fitness evaluations for particular uses. This framework served as a preliminary theoretical basis for our study, allowing us to explore how DICs manage and align data requester expectations concerning observational DQ throughout the data lifecycle—from the initial request to the final delivery. In addition to examining the procedural aspects of DUP-related DQ management, our survey aimed to uncover the underlying mechanisms through which DICs implement and communicate the fitness-for-purpose of data within research and clinical teams. Additionally, we sought to identify the standards required by each DIC for a scalable, automated solution to assess and report on data fitness-for-purpose in the MIRACUM DUP context.

### Data Analysis

We analyzed the data using the thematic analysis (TA) method suggested by Braun and Clarke [31,32]. This approach was strategically chosen to identify and rectify any potential errors in the coding process. This methodology aligns with best practices recommended for qualitative research and is supported by precedents established in similar studies [31-33]. The TA framework offers practical and meaningful steps to deeply understand the common thoughts, experiences, or behaviors [34] among the specific cohort of participants.

Following the TA framework of Braun and Clarke [32], our analysis progressed through 6 structured stages, including data familiarization, initial codes generation, theme identification, theme review, theme definition and naming, and report writing [32,34]. First, we familiarized ourselves with the material through multiple, exhaustive readings of each location’s feedback and took targeted notes. This facilitated the jotting down of early impressions. Second, we generated initial codes by manually classifying each relevant data segment. In this context, we proposed an initial coding concept (Table 1), which we created based on the vivo coding [34] (verbatim coding) method. This consisted of codes derived from the data by mainly using the language and terminology used by the study participants. This approach helped encapsulate codes reflecting the perspectives and actions expressed by the study participants. Throughout this process, there were numerous iterative discussions within the research team. Third, we used a dynamic approach to combine, compare, and analyze each of the generated code. This interpretive process enabled to inductively derive appropriate themes that have a concise and meaningful connection to the survey data. However, we ensured the themes also accurately reflected the entire data set. In the fourth step, we thoroughly reviewed these themes, ensuring that each theme has sufficient commonality, coherence, and distinctiveness to each other. In the fifth step, we assigned descriptive and accurate titles to each theme, enabling a comprehensive illustration of the key information of participants’ responses. Finally, in the sixth step, we created this manuscript as part of a TA-guided qualitative data analysis process. The condensed analytical process documentation is included in Multimedia Appendix 2. The main objective of the initial coding was to ascertain the reliability and accuracy of the framework prior to its application to a larger data set, thereby enhancing the overall integrity and validity of the research findings. Furthermore, the research team collaboratively discussed and refined the initial coding to avoid overlapping with other codes. This supported the refinement of the initial coding to the final code (see Results section). This increased both the relevance and the representativeness of the inductively generated themes and codes in light of the large information corpus provided by the participating DIC locations. The final codes are presented in the *Results* section of this paper.

We migrated the aggregated data from the confluence platform into a comprehensive Microsoft Word document for in-depth analysis (Multimedia Appendices 2 and 3). The collected data from the first survey question were analyzed using the open-source software RStudio (Posit) [35]. The scripts used for this analysis are presented in Multimedia Appendix 4.

**Table 1:** Initial coding.

Selected preliminary codes	Illustrating examples of key terms
Clinical research purposes as primary emphasis of data use projects	Clinical research, clinical trials, and observational studies
Applying the 4-eyes principle	Mutual control and the involvement of an independent person
Using overview dashboard	Data portal, integration portal, and information dash
Check for data plausibility	Plausibility checks, data verification, and data items comparisons
System comparisons	Data quality comparison between data sources and target systems
Data consistency checks	In-depth examination of data completeness, data conformity, and data correctness (including plausibility) with regard to an intended data use
Data provenance collection	Collection and documentation about where the data came from

## Results

### Overview

This study involved all 10 MIRACUM DICs including 17 participants. The greatest proportion of the participants were male (11/17, 65%), had backgrounds in computer sciences (8/17, 47%), and held a master’s degree (6/17, 35%). On average, the participants had 3.7 (SD 5.3) years of experience in assessing DQ and 1.3 (SD 1.5) years in evaluating data fitness-for-purpose. The study also revealed that most MIRACUM-affiliated DICs conduct 2 to 5 DUPs quarterly, with a single location handling up to 20 DUPs in the same timeframe. Additionally, the analysis identified 27 codes grouped into 6 themes.

### Scope of Participants

Of the 10 MIRACUM DIC sites solicited for participation, all accepted to engage in the study. In total, 17 participants agreed to respond to the survey questionnaire. Table 2 presents the demographic characteristics of the participants and shows the distribution of their years of experience in assessing both general DQ and data fitness-for-purpose.

Table 3 shows an overview of the DUP frequencies across the MIRACUM DICs. The MIRACUM-affiliated sites have undertaken an average of 5.8 (SD 6.5) DUPs on a quarterly basis, with a few sites executing up to 15 or 20 DUPs within the same timeframe. One DIC did not conduct any DUP.

**Table 2:** Overview of respondent’s metadata (N=17).

Features		Values
**Sex, n (%)**		
	Male	11 (65)
	Female	4 (23)
	Others	0 (0)
	Missing	2 (12)
**Educational background, n (%)**		
	Informatics related	8 (47)
	Statistics related	1 (6)
	Health related	2 (12)
	Others	1 (6)
	Missing	5 (29)
**Highest degree, n (%)**		
	Doctoral grade	1 (6)
	Master	6 (35)
	German “Diplom”	3 (18)
	Bachelor	1 (6)
	Vocational training	1 (6)
	Missing	5 (29)
**Years of experience with data quality assessment**		
	Mean (SD)	3.7 (5.3)
	Range	0-20
**Years of experience with observational data fitness-for-purpose assessment**		
	Mean (SD)	1.3 (1.5)
	Range	0-4

**Table 3:** Quarterly distribution of data use project frequencies across the Medical Informatics for Research and Care in University Medicine (MIRACUM) Data Integration Centers as of May 2022.

DUP^a^ frequencies		Values (n=58)
**MIRCAUM sites, n (%)**		
	MIRACUM site 1	20 (34)
	MIRACUM site 2	3 (5)
	MIRACUM site 3	15 (26)
	MIRACUM site 4	4 (7)
	MIRACUM site 5	2 (4)
	MIRACUM site 6	3 (5)
	MIRACUM site 7	4 (7)
	MIRACUM site 8	0 (0)
	MIRACUM site 9	6 (10)
	MIRACUM site 10	1 (2)
**Summary statistics**		
	Total of DUP frequencies, n (%)	58 (100)
	Mean (SD)	5.8 (6.5)
	Frequency range	0-20

^a^DUP: data use project.

### Thematic Representation of Data

#### Overview

This subsection presents a thematic representation of findings derived from responses in the MIRACUM DICs. Through a comprehensive coding process, we identified 27 distinct codes, which were subsequently organized into 6 key themes. These themes approach various relevant aspects of assessing the fitness for use of EHR data within the context of DUPs. Below is an overview of the identified themes: objectives of DUPs in MIRACUM DICs, use of heterogeneous types of data repositories, strategies for gathering DUP-specific DQ criteria, methods for evaluating the data fitness-for-purpose, existing implementations and reporting mechanisms for data fitness-for-purpose, and requirements for a scalable data fitness-for-purpose assessment solution. Results are presented below, summarized and illustrated with quotes from site feedback, aligned with the COREQ (Consolidated Criteria for Reporting Qualitative Research).

#### Objectives of DUPs in MIRACUM DICs

Most MIRACUM sites (8/10, 80%) reported that DUPs primarily engaged in clinical research, with a focus on the analysis of clinical events, the assessment of health care quality, and the development of prediction models for clinical associations. The participating sites provided detailed accounts of their research activities.

The MIRACUM site 4 underscored, for instance, the relevance of qualifying research questions and quality assessment, including “Qualifying research questions (doctoral dissertations etc.), quality assessment, proof of qualification, where so far mostly the mirror system of ORBIS serves as data repository.” Another participating site illustrated the type of clinical questions they address, by focusing on “Clinical research questions e.g. number and context data on splenectomies, context data on urological sepsis.” This indicates an in-depth examination of specific medical procedures and conditions, as observed at MIRACUM site 4.

A further narrative provided a broader perspective on the research activities, describing a research focus on

Department- and unit-specific clinical questions e.g., prediction of departmental sepsis and associations with specific treatment procedures/ICD diagnoses. Other example: patient case-based analysis of multiple clinical complications associated with specific clinical and demographic characteristics.

This comprehensive approach at MIRACUM site 9 exemplifies the depth and complexity of the clinical research being conducted, which would aim to link various clinical and demographic factors with health outcomes. These narratives collectively describe the diverse and detailed nature of the clinical research efforts within DUPs, thereby demonstrating an important commitment to improving health care quality and developing predictive models based on extensive clinical data.

#### Use of Heterogeneous Types of Data Repositories

##### Overview

In the analysis of clinical data repositories used for DUP execution, 5 prominent repositories were identified from site feedback, revealing a diverse and dynamic landscape of data management systems. These systems range from broadly used ones such as the Clinical Data Warehouse (DWH) to specialized frameworks designed to meet specific research needs or privacy considerations. This variety reflects the differing technological preferences across sites and underscores the complexity of DQ and data management in clinical research settings. The key repositories are as follows:

##### Use of Clinical DWH

The Clinical DWH emerges as the most commonly used repository for executing DUPs, as evidenced by its application in DUP-related DQA and reporting activities. In total, 3 (30%) of the 10 surveyed sites indicated that they primarily rely on the DWH for their internal research queries, quality assurance, and reporting needs. The respondents at MIRACUM site 2 and site 9 indicated that while the DWH is the primary repository for general queries, other specialized repositories, such as Informatics for Integrating Biology and Bedside (i2b2), Observational Medical Outcomes Partnership (OMOP), and Fast Healthcare Interoperability Resources (FHIR), are reserved for specific project needs, such as MI-I or MIRACUM requests. The following statements can show this:

Internal research queries, quality ensuring and reporting are mainly performed using the DWH. The i2b2/OMOP/FHIR repositories are mainly used for MI-I/MIRACUM specific requests.

Survey question 2, MIRACUM site 2

Most queries through the cDWH and i2b2 repo.

Survey question 2, MIRACUM site 9

##### Data Representation Model (i2b2, OMOP, and FHIR)

These models are notably prevalent, being used in 7 (70%) of the 10 sites. They are particularly suited to internal and cross-location DUP projects that require specific data handling or analysis frameworks. This pervasive use is corroborated by feedback from MIRACUM site 2, where i2b2 and OMOP are used for internal projects: “The i2b2/OMOP/FHIR repositories are mainly used for MI-I/MIRACUM specific requests.” This indicates the existence of a relevant and versatile framework capable of supporting a multitude of research needs.

##### ORBIS System Use

At 1 (10%) surveyed site, the ORBIS system, particularly its mirror component, is exclusively used as the primary data repository. The system supports a range of academic and quality assurance activities, such as doctoral dissertations and qualification proofs, thereby emphasizing its specialized application in academic and clinical research environments. This is presented in the following statement: “Qualifying research questions (doctoral dissertations etc.), quality assessment, proof of qualification, where so far mostly the mirror system of ORBIS serves as data repository” (Survey question 2, MIRACUM site 3).

##### OPAL DataSHIELD Framework

In total, 2 (20%) of the 10 sites surveyed indicated a preference for the OPAL DataSHIELD framework for data storage and analysis. This preference may indicate a strategic choice for environments where data privacy and security are of paramount importance. This is evidenced by the use of DataSHIELD for analysis without direct querying of data repositories, as observed at MIRACUM site 10: “Analyzing is performed via DataSHIELD, therefore no direct query in data repositories” (Indirect i2b2).

##### CentraXX Repository

The local research repository CentraXX is referenced by 1 (10%) site for the purpose of storing frequently requested data items. The respondents from MIRACUM site 6 reported that “The most frequently requested data items are stored in the local research repository CentraXX” (Survey question 2). This repository’s use serves to illustrate its role in facilitating rapid access to high-demand data, which could be of paramount importance for the efficient conduct of DUPs at the local level.

#### Strategies for Gathering DUP-Specific DQ Criteria

##### Overview

The analysis of data collection methods used by the DICs for the DUPs reveals a multifaceted approach aimed at enhancing the fitness of data through rigorous validation processes and strategic requester-provider interactions. In this subsection, we present the reported practices to ensure the alignment and quality of data for DUPs.

##### Detailed Request Submission

A noteworthy observation is that a considerable proportion of DICs (4/10, 40%) emphasize the importance of detailed and precise data requests. This approach serves as a preemptive measure to ensure that the data delivered are aligned with the needs of the requester. In the event of discrepancies between the requested and provided data, the aforementioned sites use a postprocessing step to rectify any misalignments. This process is captured by a statement from one of the survey respondents:

During the data request, we advise that the requested data should be described as fine-grained and exact as possible. If the data provided does not match the request, a “post-processing” process will be initiated.

Survey question 3, MIRACUM site 1

##### Participatory Discussion for Data Validation

Half of the surveyed sites (5/10, 50%) use participatory discussions between data providers and requesters as a core strategy for data validation. These discussions aim to identify and mitigate any factors that might reduce the quality of the data, such as issues with free text information or the specific documentation practices of the data-providing institution. The respondents noted that:

The requested data are usually discussed at least once with the requester and quality-reducing aspects are worked out together, e.g., free text information, documentation practice in the respective data-providing institution (usually the requester comes from the same institution and knows it very well).

Survey question 3, MIRACUM site 3

##### Quality Requirements Gathering

A collection of DQ requirements is conducted at a minority of the sites (1/10, 10%), with the use of a feasibility or data request form. The form is completed by both data requesters and internal data request administrators in order to gather specific DQ requirements. The aforementioned process is described as follows:

Project-related data quality requirements are gathered using a Feasibility Request (FR) form completed by the data requester & internal data request administrator.

Survey question 3, MIRACUM site 9

However, 2 (20%) of the 10 sites did not apply any approach to gather the DUP-related DQ requirements.

#### Methods for Evaluating the Data Fitness-for-Purpose

##### Overview

In terms of applied methods to assess whether the DIC data suite is for the carrying out of various DUPs, 3 approaches were revealed.

##### Four-Eyes Principle

This method, which was observed at 3 (30%) of the 10 sites, places an emphasis on ensuring that the data are fit for the intended purposes through mutual control and content validation. The fundamental principle is straightforward: before any data are provided or issued. It is subjected to scrutiny by at least 2 individuals, thereby enhancing both accuracy and reliability.

The MIRACUM site 3 highlighted the development of a metadata repository–supported DQA tool, indicating that this approach is being further systematized: “Mutual control before issue/provision (4-eyes principle), an MDR-supported DQA tool is under development.”

Similarly, MIRACUM site 9 provided further insight into the practical application of this principle, describing a 2-tier validation process as follows:

4-eyes principle: Content validation of the queries by a second data scientist (possibly also with a separate query), so that it is ensured that the query actually does what it is supposed to do. Content-related plausibility control of the results from the query through medical colleagues.

##### Comparison of Data Values Distribution

This approach, used by 2 (20%) of the 10 sites, validates data by contrasting the distribution of data values from different systems. The MIRACUM site 2 provided an illustrative example of this method by comparing the hit ratios generated by independent systems: “For project-specific validation, comparison of hit ratio from different systems created by an independent person.” This process not only identifies discrepancies or anomalies but also serves to reinforce the integrity of the data through independent verification.

##### Data Consistency Checks

The consistency checks are used by 2 (20%) of the 10 sites to verify the appropriateness of data formats, types, and variable associations. The MIRACUM site 10 exemplifies this process by using DataSHIELD to verify data formats and the number of variables prior to initiating analyses: “Verification of the data format or type, the number of variables via DataSHIELD before the analyses.” This step could be of paramount importance in ensuring that the data meet the requisite standards for subsequent analytical procedures. It might serve as a foundational check that prevents the propagation of errors in data handling and analysis.

#### Existing Implementations and Reporting Mechanisms for Data Fitness-for-Purpose

##### Overview

The following measures were identified to technically implement and report fitness-for-purpose of clinical data.

##### Data Requester Feedback and Adjustment Process

At a majority of the study sites (6/10, 60%), the implementation of a feedback mechanism that involves data requesters is of high importance. Here, data requester feedback is systematically gathered and analyzed. In light of this input, the queries used to select data undergo adjustments and validations in accordance with the 4-eyes principle. This process is illustrated by a representative from MIRACUM site 9:

Then the data request administrator, who goes through the data to be delivered together with the data requester, delivers the data. In case of change requests/incorrect quality in the data, the data selection queries are adjusted and validated again via the 4-eyes principle, and documented.

##### Feedback Loop and Quality Control

In 2 (20%) of the 10 surveyed sites, a continuous feedback loop is established among the data requester, data request administrator, internal data scientists, and the data transfer office. This iterative process is highly relevant for the refinement of DQ prior to final delivery. As described by MIRACUM site 9, the cycle involves a series of checks and rechecks:

This results in the feedback cycle: data requester => data request administrator => internal data scientists => data request administrator => data requester. Only in case of a complete match (from the data requester’s perspective) the final data delivery takes place.

##### Reporting Quality Using Dashboard

The use of technological tools to report on DQ was observed in 2 (20%) sites. Specifically, these sites use an overview dashboard within a self-developed data integration portal. This tool provides a streamlined and transparent view of DQ metrics, which can be essential for ongoing assessment and improvement. A representative from MIRACUM site 7 described the utility of the dashboard as follows: “Overview dashboard in the self-developed data integration portal,” indicating a technology-driven approach to quality reporting.

##### Requirements for a Scalable Solution for the Data Fitness-for-Purpose Assessment

Table 4 summarizes the requirements gathered during the survey including some exemplifying quotes. The MIRACUM DICs emphasized diverse key attributes for the development of a data fitness-for-purpose assessment tool. These include flexibility, understandability, practicability, and extendibility in terms of usability. From a technical perspective, there was a preference for dashboard implementation, system comparison features, data consistency checks, and ensuring uniformity in the FHIR profiles. Additionally, the consideration of data provenance was mentioned as an important feature in assessing fitness-for-purpose.

**Table 4:** Summary of requirements for implementing a data fitness-for-purpose assessment tool.

Requirement dimension and key attributes			DICs^a^ (n=10), n (%)	Illustrating quotes
**Usability**				
	Flexibility	1 (10)		“Flexible organization of the DQ system” [Survey question 6, MIRACUM^b^ site 1].
	Understandability	2 (20)		“Understandability for the clinician and data scientist/statistician Fitness-for-use dashboard” [Survey question 6, MIRACUM site 2].
	Practicability	1 (10)		“Generally enough that it can be used in every DIZ and for every request. It should be pragmatic and easy to understand, so that it can always be used as a basic tool and its benefits are seen equally by all parties (data provider, data supplier, data requester)” [Survey question 6, MIRACUM site 3].
	Extendibility	1 (10)		“In the short term, it is limited to the essentials to be able to use it and gain experience. In the long term, it may even be possible to modularize it and thus use it only in parts” [Survey question 6, MIRACUM site 3].
**Technical and functionalities**				
	Dashboard implementation	3 (30)		“Implementation of a dashboard” [Survey question 6, MIRACUM site 1].
	System comparison	3 (30)		“Complete non-interactive integration of the DQ process as an operation within the data pipelines for complete monitoring of the mapping of source and target systems with automatic machine-readable report generation (no PDF)” [Survey question 6, MIRACUM site 7].
	Data consistency checks	3 (30)		“Mapping and automation of DQ checks based on the specific data quality metrics Data completeness: are there enough patients at the DIZ site to carry out the planned projects Data plausibility: formulation & automation of general-transferable plausibility checks (e.g., no readmission after a death, ...) that could affect the outcomes of most DRs Data conformity: uniform mapping and verification of conformity of ICD, OPS, LOINC codes, and adequate reporting in the systematics” [Survey question 6, MIRACUM site 9].
	FHIR^c^ profiles uniformity	3 (30)		“Uniform FHIR profiles across MIRACUM partners” [Survey question 6, MIRACUM site 10].
	Data provenance collection	1 (10)		“Structured Provenance Documentation: where did the data come from, what processing steps were performed on the data up to the time of data delivery, Are there changes to the data that may represent a potential impact on the planned data use project?” [Survey question 6, MIRACUM site 9].

^a^DIC: Data Integration Center.

^b^MIRACUM: Medical Informatics in Research and Care in University Medicine.

^c^FHIR: Fast Healthcare Interoperability Resources.

## Discussion

### Principal Findings

#### Overview

This investigation examined the practices surrounding the fitness-for-purpose of observational health care data within MIRACUM DICs. It revealed both strengths and areas needing improvement in current approaches compared to established evidence. It also highlighted the active engagement of MIRACUM DICs in assessing data suitability for clinical research, using a diverse array of data repository infrastructures. However, the findings also underscored an important variation in methods for DQA and a general lack of standardization, suggesting the need for a more harmonized approach to enhance the accuracy and reliability of clinical data for specific secondary use purposes. Practical requirements were identified to support future implementation studies toward automating the observed processes.

#### Addressing Fitness-for-Purpose of Observational Health Care Data

Our study set out to examine the extent to which MIRACUM DICs address the fitness-for-purpose of observational health care data and the ways in which these approaches differ from existing benchmarks. On the one hand, the demographic data suggest that the majority of participating MIRACUM DIC DQ experts have a computer science background and the prevalent academic qualification was a master’s degree. This is indicative of the technological proficiency of the participants about data handling and engineering, but the participants may lack specific clinical or statistical expertise. This could be required for accurately assessing the suitability of clinical data for secondary use purposes. On the other hand, it is apparent that the MIRACUM DICs are actively engaged in fitness-for-purpose assessment of data, particularly for clinical research questions ranging from clinical event investigations to complex prediction models. This reflects the substantial amount of evidence [3,5,8,9,12] achieved since the inception of the German MI-I, implying a potential of fitness-for-purpose in DIC data, which should be further investigated based on the DIC data–driven research question. Interestingly, various repositories are used, including Clinical DWH, i2b2, OMOP, FHIR, ORBIS system, and OPAL DataSHIELD framework, indicating the use of diversified data infrastructure. However, there is a lack of harmonization and standardization in terms of strategies for assessing the suitability of DIC data with methods including the 4-eyes principle, system comparisons, and data consistency checks.

Compared to existing evidence, carrying out data consistency checks by the DICs nuancedly aligns with the recommendations of Kahn et al [22]. This process helps the MIRACUM sites in using the existing MIRACUM DQA tool to determine whether the integrated DIC data across the source and target databases [14,24] adhere to specified standards (conformance), whether certain data elements or values are sufficient (completeness), or if they are accurately reported (plausibility). In contrast, implementing the items from the 3×3 DQA guideline proposed by Weiskopf et al [21] was barely observable. This approach recommends specific DUPs by using particular methods (eg, rate of data regularity as proposed by Sperrin et al [36]) to assess DQ. For instance, this involves examining whether information regarding patients, data variables, and timeframe are completely, correctly, and currently integrated. This enables the research or clinical team to transparently determine the fitness-for-purpose of the data delivered by DICs. Automating such a process would enhance the integration of much more evidence, ensuring DQ for executing DUPs within the MIRACUM DICs. Therefore, the investigation of Razzaghi et al [37] could be seen as an initial roadmap toward a technical implementation. This describes a framework operationalizing such a fitness-for-purpose assessment process, with a particular focus on assessing clinical diagnosis or care. Furthermore, this should be evaluated in a closed association with DQ principles (outlier detection, plausibility, etc), as reported in various existing recommendations [20-22,38].

#### Key Requirements for Automating the Assessment Process

Our second research question focused on identifying the essential requirements for automating the assessment of fitness-for-purpose of EHR data within MIRACUM DICs. The TA revealed that from the perspective of the MIRACUM DICs, a scalable system for assessing the suitability of clinical data should be flexible, easily understandable, practicable, and modularizable (able to be extended in modules). However, the DICs expressed a need for user-friendly dashboards, facilitating an automated performance of system comparisons, data consistency checks, and data provenance collection in DUPs. These practice-oriented expectations adhere to the definition of fitness-for-purpose proposed by Girman et al [23], which considers assessing the availability of relevant data and the data provenance as key points for capturing fitness-for-purpose. A prior study by Gierend et al [15] explored the current status of provenance collection, which may present straightforward support in implementing this approach at the MIRACUM DIC level. However, the heterogeneity in the used data repositories at the DICs revealed that any automated solution would need to be repository agnostic or interoperable with multiple type of databases. In addition, the strategies for gathering of DUP-specific DQ criteria included consistent data description by requesters and participatory discussion between data providers and requesters. This suggests that an automated solution would require incorporating a flexible but stringent set of DQ criteria or functions, such as suggested by Weiskopf et al [20,21], Razzaghi et al [37], or Schmidt et al [38], that can be adjusted based on dynamic inputs. Finally, common approaches for assessing the fitness-for-purpose included the 4-eyes principle, comparison of data value distribution, and data consistency checks. These DQ verification processes suggest the need to incorporate robust DQA mechanisms into automation. These mechanisms could potentially use machine learning or rule-guided algorithms similar to the machine learning–supported DQA framework developed by Mark et al [39]. By considering these diverse aspects, we can consistently streamline the assessment of the fitness-for-purpose of clinical data, benefiting both research and clinical applications.

### Derivation of a Roadmap Framework for Fitness-for-Purpose Assessment

#### Overview

**Figure 1:**
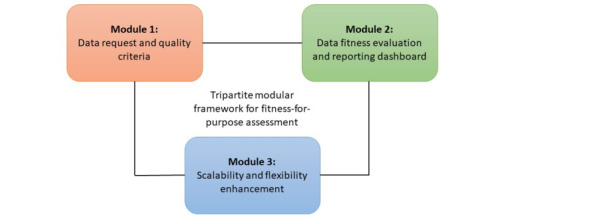
Tripartite modular framework for fitness-for-purpose assessment.

Based on the findings discussed earlier, a guiding framework for an automated solution for assessing the fitness-for-purpose of EHR at the MIRACUM DICs was elaborated and structured into 3 modules (Figure 1). These modules have been designed to be pragmatically implementable and closely aligned with the survey insights to facilitate a smooth transition into practical application.

#### Data Request and Quality Criteria Module

This module should centralize and streamline the data requests and collection of DUP-specific DQ criteria. This module should include a user interface for data requesters to input their data requirements (eg, study fit criteria) and fitness-for-purpose criteria (eg, based on DQ rules from the 3×3 framework [21]).

#### Data Fitness and Reporting Dashboard Module

This module incorporates mechanisms for data fitness assessment reflecting the need for evaluating the data fitness for specific DUPs. It should use the fitness-for-purpose criteria collected from module 1 to perform data accuracy evaluation, alongside functionalities for comparing data values across different systems (eg, i2b2, OMOP, and FHIR). Furthermore, this module should feature a decision-supporting dashboard for visualizing these assessments by providing an overview of the data’s fitness-for-purpose, which may facilitate structured documentation of DQ assurance tasks into the data management plans of DUPs.

#### Scalability and Flexibility Enhancement Module

This module is dedicated to the technical aspects of items from modules 1 and 2, addressing the requirements for a scalable solution, including the implementation of a user interface and dashboard, system comparison features, data consistency and provenance checks, and uniformity in FHIR profiles. Moreover, it should be implemented under consideration of usability requirements expressed by the DICs.

Each module was elaborated to address specific aspects identified in the survey to ensure a comprehensive and user-friendly solution for the automated DQ or fitness-for-purpose tool. This approach should not only facilitate the implementation process but also improve the efficiency and effectiveness of DQ and fitness-for-purpose checks across all MIRACUM DICs.

### Strengths and Limitations

The strengths of this investigation lie in its comprehensive data collection and analysis. Using established approaches, such as the TA-based framework of Braun and Clarke [31,32], we provided a holistic view of the current state of the assessment of fitness-for-purpose observational EHR data at the MIRACUM DICs. Given the importance of reflexivity in qualitative research, as discussed by Braun and Clarke [32], we acknowledge our theoretical stance toward a constructivist approach, where knowledge is viewed as a construct rather than discovery. This perspective informed our analysis to interpret the data through a lens that considers both the factual and the contextual dimensions conveyed by the participants. As such, our survey questions, detailed in Textbox 1, were crafted not only to gather empirical information but also to probe deeper into the implications and the perceived effectiveness of DQ practices. Furthermore, the comprehensive examination of all MIRACUM DICs, illustrated diversified ways of conducting secondary use of EHR data and fitness-for-purpose assessments, highlighting additional proficiency.

However, our study also presents some limitations. The study cohort was restricted to the MIRACUM consortium. Expanding the research to include other MI-I consortia and DICs not affiliated with the MI-I project would enhance the sample’s representativeness and the generalizability of the earned findings.

### Implications for Future Research and Practice

To ensure that DIC data are suitable for research and clinical applications, it is crucial for MIRACUM DICs to align their DQA processes with established best practices and recommendations. Future research should aim to investigate the correlation adhering to the recommendations with the successful conduction of DUPs by evaluating whether clinical research questions (eg, cross-location analysis of comorbidities [25]) were more consistently addressable. For research practice, there is an accentuated need to develop automated and scalable solutions to assess the fitness-for-purpose of EHR DIC data, and our study provides the foundational insights to drive this technological advancement. Importantly, the solution could be based on the proposed tripartite modular framework. This could ease the integration into existing infrastructures of MIRACUM and eventually MI-I DICs, taking into account the key requirements identified by this investigation.

### Conclusions

While the MIRACUM DICs focus on assessing the quality of clinical data for DUP conduction, there is a relevant variation in the used methods and requirements to automate these processes. With the increasing volume and complexity of health data, having a standardized and scalable solution to automate the assessment of fitness-for-purpose is essential to maintain the integrity of research and clinical practice. Follow-up investigations should be directed toward building systems guided by evidence-based practices that should be customized to the specific needs and circumstances of MIRACUM and MI-I DICs.

## Appendix

Multimedia Appendix 1 Instructions for sites participation at the survey.

Multimedia Appendix 2 Documentation of qualitative analysis of survey feedback data.

Multimedia Appendix 3 Survey feedbacks from the participating Medical Informatics for Research and Care in University Medicine sites (deidentified).

Multimedia Appendix 4 R-Project to perform the visualization of data use project frequencies.

## Abbreviations

COREQ: Consolidated Criteria for Reporting Qualitative Research
DIC: Data Integration Center
DQ: data quality
DQA: data quality assessment
DUP: data use project
DWH: Data Warehouse
EHR: electronic health record
FHIR: Fast Healthcare Interoperability Resources
GESIS: Society of Social Science Infrastructure Institutions
i2b2: Informatics for Integrating Biology and Bedside
MI-I: Medical Informatics Initiative
MIRACUM: Medical Informatics in Research and Care in University Medicine
OMOP: Observational Medical Outcomes Partnership
TA: thematic analysis

## Data Availability

The qualitative data collected and analyzed during this study are available in Multimedia Appendices 2 and 3. Furthermore, the R-based script used to plot the data use project execution frequency is available as well in Multimedia Appendix 4.
