# Supplementary material for: Evaluating and Enhancing the Fitness-for-Purpose of Electronic Health Record Data: Qualitative Study on Current Practices and Pathway to an Automated Approach Within the Medical Informatics for Research and Care in University Medicine Consortium
Source: JMIR Med Inform. 2024 Aug 19;12:e57153. doi: 10.2196/57153 (PMC11369535; doi:10.2196/57153)
Supplement: Multimedia Appendix 1 [file medinform_v12i1e57153_app1.pdf]

Instructions for sites participation at the survey:

Background Information:

- For a requirement-oriented development of a cross-site data quality systematic, which is to support the collection and analysis of the DQ related to specific data use projects across the Data Integration Centers (DICs), an as-is analysis is of high relevance in advance.
- On the appropriate Confluence subpage, a documentation of the survey feedback from your site will be conducted, about how you could achieve the collection and interpretation of the data quality related to specific data use projects so far (Fitness-for-Purpose).
- Furthermore, their (specific) requirements and expectations for a cross-site harmonized Fitness-for-Purpose DQ systematic are also collected and documented.

Instructions for answering the survey:

- Answering the questionnaire can take up to approx. 30 min.
- Please answer punctually and include more meaningful examples of possible answers
- Please remember to complete the header information accordingly before the release. Set deadline: 06.05.2022
- For any questions, please contact Gaetan Kamdje Wabo
