# Supplementary material for: Evaluating and Enhancing the Fitness-for-Purpose of Electronic Health Record Data: Qualitative Study on Current Practices and Pathway to an Automated Approach Within the Medical Informatics for Research and Care in University Medicine Consortium
Source: JMIR Med Inform. 2024 Aug 19;12:e57153. doi: 10.2196/57153 (PMC11369535; doi:10.2196/57153)
Supplement: Multimedia Appendix 2 [file medinform_v12i1e57153_app2.pdf]

Qualitative content analyses of survey results

Question 1: On average, how many data requests or local data use projects are handled at your site DIC per quarter?

Table S1: Results from Question 1

Sites-ID	Feedbacks	Counts
1	20 (We expect significantly more requests in the following quarter, as our data catalog is still in the publication mechanism. The 20 therefore refer to requests that have already been generated even though the data catalog has not yet been published.)	20
2	Our UAC processes approximately 3 data use requests per quarter.	3
3	approx. 15	15
4	An average of 4 requests per quarter	4
5	approx 2 per quarter	2
6	3 (estimation)	3
7	Q1/2022: 4 projects<br>Quarterly very irregular<br>Total since project start 35	4
8	Officially 0, as not yet approved by data protection	0
9	approx. 6<br>in Q1/2022 n=7	6
10	One data request per quarter on average	1

Question 2: Which contents are mostly in focus in your local data use projects (e.g.: care evaluation in transfusion medicine, etc.), and which data repositories are most frequently queried in this context (i2b2, OMOP, FHIR, ...)?

Table S2: Screening of feedbacks from Question 2

Sites-ID	**Feedbacks**	**Inductive code generation for Question 2**
**1**	clinical research<br>Self-research by physicians<br>Currently, the clinical data repository is the most queried.<br>FHIR is in the process of implementation.	DUP are mostly focused on Clinical research purpose<br>Clinical DWH is the most queried data repository
**2**	Internal research queries, quality ensuring and reporting are mainly performed using the DWH. The i2b2/OMOP/FHIR repositories are mainly used for MI-I/MIRACUM specific requests.	DUP are mostly focused on Clinical research purpose<br>Clinical DWH is the most queried data repository<br>I2b2 or OMOP or FHIR-databases for internal or for MI/MIRACUM specific requests
**3**	Qualifying research questions (doctoral dissertations etc.), quality assessment, proof of qualification, where so far mostly the mirror system of ORBIS serves as data repository; the mentioned i2b2/OMOP/FHIR mostly play a role only for MI-I-/MIRACUM-specific queries.	Mirror system of ORBIS as source data repository<br>DUP are mostly focused on Clinical research purpose<br>I2b2 or OMOP or FHIR-databases for internal or for MI/MIRACUM specific requests
**4**	Lab values and diagnosis within specialties: Neurology, Urology, Pneumology, Internal Medicine.<br>i2b2, omop, cdr (internal projects), fhir<br>the analysis is performed on OPAL/DataSHIELD	DUP are mostly focused on Clinical research purpose<br>OPAL/DataSHIELD as data analysis system<br>I2b2 or OMOP or FHIR-databases for internal or for MI/MIRACUM specific requests
**5**	Clinical research questions e.g. number and context data on splenectomies, context data on urological sepsis.<br>Target repository is i2b2 and FHIR	DUP are mostly focused on Clinical research purpose<br>I2b2 or OMOP or FHIR-databases for internal or for MI/MIRACUM specific requests
**6**	Query only possible directly at Data Integration Center (DIC); DIC extracts the data and makes it available for use. The most frequently requested data items are stored in the local research repository CentraXX. This is followed by requests for FHIR data from the national projects.	I2b2 or OMOP or FHIR-databases for internal or for MI/MIRACUM specific requests Storage of requested data items into CentraXX
**7**	Case numbers for diagnoses/treatment procedures<br>Requests by all specialties<br>i2b2 (with Apache Superset as interface since 2022)<br>fhir-server	DUP are mostly focused on Clinical research purpose<br>I2b2 or OMOP or FHIR-databases for internal or for MI/MIRACUM specific requests

**8**	n/a	
**9**	Department- and unit-specific clinical questions<br>e.g., prediction of departmental sepsis and associations with specific treatment procedures/ICD diagnoses.<br>Other example: patient case-based analysis of multiple clinical complications associated with specific clinical and demographic characteristics.<br>Most queries through the cDWH and i2b2 repo.	DUP are mostly focused on Clinical research purpose<br>Clinical DWH is the most queried data repository<br><br>I2b2 or OMOP or FHIR-databases for internal or for MI/MIRACUM specific requests
**10**	Mainly retrospective data analysis in pulmonology.<br>Analyzing is performed via DataSHIELD, therefore no direct query in data repositories. (Indirect i2b2)	DUP are mostly focused on Clinical research purpose<br>OPAL/DataSHIELD as data analysis system

Table S3: Resulting themes and Codes from the Question 2

**Themes (From a data content-based inductive process)**	**Codes (From a data content-based inductive process)**	**Counts**
Purpose of Data Use Projects (DUPs)	DUPs are mostly focused on Clinical research purpose	8
Research infrastructure and repositories	Clinical DWH is the most queried data repository for DUP	3
	I2b2 or OMOP or FHIR-databases for internal or for MI/MIRACUM specific requests	7
	Mirror system of ORBIS as source data repository	1
	OPAL/DataSHIELD as data analysis system	2
	Storage of requested data items into CentraXX	1

Question 3: How are data use project-specific data quality (DQ) requirements collected from the perspective of data requesters at their DIC?

Table S4: Screening of feedbacks from Question 3

Sites-ID	**Feedbacks**	**Inductive Codes generation for Question 3**
**1**	During the data request, we advise that the requested data should be described as fine-grained and exact as possible. If the data provided does not match the request, a "post-processing" process will be initiated.	The data requesters provide an explicit description of expected data
**2**	In general, the heads of the projects contact the transfer office/UAC office and clarify which data can be extracted and which variables are useful for a scientific evaluation and what should be considered (specific conventions/documentation)	The data requesters provide an explicit description of expected data<br>Data validation through a discussion between data provider and data requester
**3**	I am not sure exactly how the question is meant. In any case, the requested data are usually discussed at least once with the requester and quality-reducing aspects are worked out together, e.g. free text information, documentation practice in the respective data-providing institution (usually the requester comes from the same institution and knows it very well).	Data validation through a discussion between data provider and data requester
**4**	Is not collected.	No collection of DQ-requirements
**5**	In interactive discussion with the researchers.<br>Environment at the moment still too heterogeneous for a standardized approach	Data validation through a discussion between data provider and data requester
**6**	In personal conversation during consultation.	Data validation through a discussion between data provider and data requester
**7**	manual explorations of the data with requesters and providers<br>100% correct data quality is assumed	Data validation through a discussion between data provider and data requester
**8**	n/a	No collection of DQ-requirements
**9**	Project-related data quality requirements are gathered using a Feasibility Request (FR) form completed by the data requester & internal data request administrator.	Usage of a Feasibility or Data Request form<br>The data requesters provide an explicit description of expected data

	Documentation of the intended cohort property (in terms of expected minimum cohort size,...) and project specifications (e.g. inclusion and exclusion criteria) takes place there	
**10**	These are additionally described in the project proposal under the item "Data description".	The data requesters provide an explicit description of expected data

Table S5: Resulting themes and Codes from the Question 3

**Themes (From a data content-based inductive process)**	**Codes (From a data content-based inductive process)**	**Counts**
Collection of DQ-requirements	The data requesters provide an explicit description of expected data	4
	Data validation through a discussion of data provider with the data requester	5
	No collection of DQ-requirements	2
	Usage of a Feasibility or Data Request form	1

Question 4: In addition to the current MIRACUM DQA tool, what tools or technical approaches do you employ for data use project-specific data quality assessment?

Table S6: Screening of feedbacks from Question 4

Sites-ID	**Feedbacks**	**Inductive subtheme generation for Question 4**
**1**	An initial concept of completeness of data elements is under review and will be implemented in Q3 2022	No working solution in parallel to the MIRACUM-DQA tool
**2**	For project-specific validation, comparison of hit ratio from different systems created by an independent person: e.g. separate i2b2 SQL queries compared to FHIR/staging area/DWH queries, etc. Before data delivery/provision, mutual control (DIC internal as well as with clinicians) and official release of results by the head of the transfer office.	Comparison of data value distribution from different systems<br>Applying the 4-eyes-principle
**3**	Mutual control before issue/provision (4-eyes principle), an MDR-supported DQA tool is under development	Applying the 4-eyes-principle
**4**	Simple site-specific count comparison of identical SQL content on CDR and source DB is established.	
**5**	Resource-specific tracking of the datapath based on a unified system of FHIR business identifiers.	Comparison of data value distribution from different systems
**6**	no further tools in addition to MIRACUM DQA-Tool.	No working solution in parallel to the MIRACUM-DQA tool
**7**	n/a	No information
**8**	n/a	No information
**9**	4-eyes principle:<br>Content validation of the queries by a second data scientist (possibly also with a separate query), so that it is ensured that the query actually does what it is supposed to do.<br>Content-related plausibility control of the results from the query through medical colleagues.	Applying the 4-eyes-principle
**10**	Formless communication to the transfer office of the DIC	Communication with the DIC transfer office

Table S7: Resulting themes and Codes from the Question 4

Themes (From a data content-based inductive process)	Codes (From a data content-based inductive process)	Counts
Current approaches for fitness-for-use assessment	No working solution in parallel to the MIRACUM-DQA tool	2
	Applying the 4-eyes-principle	3
	Comparison of data values distribution from different systems	2
	Communication with the DIC transfer office	1
	No information	2

Question 5: What measures are taken at your location to communicate with data requesting sites about the quality of provided data for the intended purpose, so that data requesters have opportunities to estimate the fitness of the data to complete the intended project?

Table S8: Screening of feedbacks from Question 5

Sites-ID	**Feedbacks**	**Inductive code generation for Question 5**
**1**	Creation of a transfer office. The transfer office communicates with the data requesting offices. After data provision, the transfer office inquiries about the satisfaction / suitability of the data with the data requesting office. After checking with the data requester site, this consults with the transfer office. If there are deficiencies in the quality of the data, the transfer office forwards this to the architects of the data. They contact the data requesting office directly in order to work out solutions together.	Advice and Collection of data requester feedbacks<br>Feedback loop: data requester staff- transfer office - internal data providing staff - transfer office - data requester staff
**2**	Conduct feasibility study<br>Communicate mid-term results	Advice and Collection of data requester feedbacks
**3**	This is done in direct dialog with the requester. (see also 3)	Advice and Collection of data requester feedbacks
**4**	The DIC advises the data requesters individually. So far, there are only a few projects in which the DIC was not scientifically represented.	Advice and Collection of data requester feedbacks
**5**	Overview dashboard in the self-developed data integration portal	Usage of an overview dashboard
**6**	Not relevant yet	No information
**7**	Scope of the core data set vs. expectations in the context of a consultation.<br>Feasibility queries<br>Comparison with known data from the hospital vs. data set together with requesters<br>Provision of a data dashboard for own queries by requesters	Usage of an overview dashboard<br>Check for data consistency
**8**	n/a	No information
**9**	Delivery of the data with involvement of the data requesters<br><br>First, the feasibility request determines to what extent the number of patients suitable for the planned project is available in sufficient amount	Advice and Collection of data requester feedbacks<br><br>Feedback loop: data requester staff- transfer office - internal data providing staff - transfer office - data requester staff

	Then the data are delivered by the data request administrator, who goes through the data to be delivered together with the data requester.<br>In case of change requests/incorrect quality in the data, the data selection queries are adjusted and validated again via the 4-eyes principle, and documented<br><br>This results in the feedback cycle: data requester => data request administrator => internal data scientists => data request administrator => data requester<br><br>Only in case of a complete match (from the data requester's perspective) the final data delivery takes place.	
**10**	Plausibility check of the provided data together with researchers (physicians) before using the data for the analysis.<br><br>Use of the uniform data dictionary (metadata).<br><br>Verification of the data format or type, the number of variables via DataSHIELD before the analyses.<br><br>If it detects inconsistencies in the research data, it will cross-check them with the source system and identify problems	Check for data consistency<br>Advice and Collection of data requester feedbacks

Table S9: Resulting themes and Codes from the Question 5

**Themes (From a data content-based inductive process)**	**Codes (From a data content-based inductive process)**	**Counts**
Communication measures	Advice and Collection of data requester feedbacks	6
	Feedback loop: data requester staff- transfer office - internal data providing staff - transfer office - data requester staff	2
Technical measures	Usage of an overview dashboard	2
	Check for data consistency	2

Question 6: What would be their expectations/requirements for a fitness-for-use cross-site DQ framework that you could adopt in the future to measure DQ related to their data use projects?

Table S10: Screening of feedbacks from Question 6

Sites-ID	**Feedbacks**	**Inductive code generation for Question 6**
**1**	Implementation of a dashboard	Dashboard Implementation
**2**	Flexible organization of the DQ system<br>Locally assessed DQ compared to sites<br>Integrate project-specific data plausibility<br>Understandability for the clinician and data scientist/statistician<br>Fitness-for-use dashboard	Flexibility<br>System comparisons<br>Data consistency checks<br>Understandability<br>Dashboard Implementation
**3**	Generally enough that it can be used in every DIZ and for every request. It should be pragmatic and easy to understand, so that it can always be used as a basic tool and its benefits are seen equally by all parties (data provider, data supplier, data requester). In the short term, it is limited to the essentials to be able to use it and gain experience. In the long term, it may even be possible to modularize it and thus use it only in parts.	Understandability<br>Extendibility<br>Practicability

**4**	Graphical representation over time (gaps, leaps in values).	Dashboard Implementation
**5**	Integration of the already used resource-specific tracking of the datapath based on a unified system of FHIR business identifiers into the DQ system.	System comparisons<br><br>FHIR profiles Uniformity
**6**	These cannot yet be definitively determined	No information
**7**	Complete non-interactive integration of the DQ process as an operation within the data pipelines for complete monitoring of the mapping of source and target systems with automatic machine-readable report generation (no PDF)<br><br>Automated comparison of previous reports (in the context of performed developments or updates)	System comparisons
**8**	Completeness<br>Plausibility<br>Currentness	Data consistency checks
**9**	Provision of a uniform template for documenting DQ and possibly also data requestor feedbacks in the context of project-related data deliveries across the DIZs<br><br>Mapping and automation of DQ checks based on the specific data quality metrics<br>• Data completeness: are there enough patients at the DIZ site to carry out the planned projects<br>• Data plausibility: formulation & automation of general-transferable plausibility checks (e.g., no readmission after a death, ...) that could affect the outcomes of most DRs<br>• Data conformity: uniform mapping and verification of conformity of ICD , OPS, LOINC codes, and adequate reporting in the systematics<br>Structured Provenance Documentation:<br>• where did the data come from,	Data consistency checks<br><br>FHIR profiles Uniformity<br>Data Provenance collection

	• what processing steps were performed on the data up to the time of data delivery,<br>• Are there changes to the data that may represent a potential impact on the planned data use project?<br>FHIR as a single target repository for the data requests (also needs to be coordinated across DIZ).<br>Inclusion of i2b2 and OMOP as additional repositories depending on whether a specific repo is preferred/specified by the data request.	
**10**	Uniform FHIR profiles across MIRACUM partners.<br>Standardization of LOINC mapping<br>Uniform measurement units	FHIR profiles Uniformity

Table S11: Resulting themes and Codes from the Question 6

Themes (From a data content-based inductive process)	Codes (From a data content-based inductive process)	Counts
Usability-related requirements for a fitness-for-use tool	Flexibility	1
	Understandability	2
	Practicability	1
	Extendibility	1
Functionalities-related requirements for a fitness-for-purpose tool	Dashboard Implementation	3
	System comparisons	3
	Data consistency checks	3
	FHIR profiles Uniformity	3
	Data Provenance collection	1

Table S12: Summary of the finalized themes and Codes from the inductive generation system

Themes	Codes
Objectives of DUPs in MIRACUM DICs	DUPs are mostly focused on Clinical research purpose
Utilization of heterogeneous types of data repositories	Clinical DWH is the most queried data repository for DUP
	I2b2/OMOP/FHIR for MI-I/MIRACUM specific requests
	Use of i2b2/OMOP/FHIR for internal projects
	Mirror system of ORBIS as source data repository
	OPAL/DataSHIELD as data analysis system
	Storage of requested data items into CentraXX
Strategies for gathering DUP-specific data quality criteria	The data requesters provide an explicit description of expected data
	Data validation through a discussion with the data requester
	No collection of DQ-requirements
	Usage of a Feasibility or Data Request form
Methods for evaluating the data Fitness-for-Purpose	No working solution in parallel to the MIRACUM-DQA tool
	Applying the 4-eyes-principle
	Comparison of data values distribution from different systems
	Communication with the DIC transfer office
Existing implementations and reporting mechanisms for data Fitness-for-Purpose	Advice and Collection of data requester feedbacks
	Feedback loop: data requester staff- transfer office - internal data providing staff - transfer office - data requester staff
	Usage of an overview dashboard
	Check for data consistency
Requirements for a scalable Data-Fitness-for-purpose assessment solution	Flexibility
	Understandability
	Practicability
	Extendibility
	Dashboard Implementation
	System comparisons
	Data consistency checks
	FHIR profiles Uniformity
	Data Provenance collection
