# Supplementary material for: Evaluating and Enhancing the Fitness-for-Purpose of Electronic Health Record Data: Qualitative Study on Current Practices and Pathway to an Automated Approach Within the Medical Informatics for Research and Care in University Medicine Consortium
Source: JMIR Med Inform. 2024 Aug 19;12:e57153. doi: 10.2196/57153 (PMC11369535; doi:10.2196/57153)
Supplement: Multimedia Appendix 4 [file medinform_v12i1e57153_app4.zip › analysis/graph_generation_code.nb.html]

Graphical Analysis from survey data


Code 

- Show All Code
- Hide All Code
- Download Rmd

# Graphical Analysis from survey data


```
library(readr)
library(tidyverse)
library(haven)
library(ggplot2)
```


```
average_datarequests <- read_csv("average_datarequests.csv")
View(average_datarequests)


average_datarequests <- transform(average_datarequests,
                                  variable=reorder(locations, -freq) )
```


```
# Set the custom color
custom_color <- "#E066FF"

# Perform the ggplot visualization 
# Saving the outcome into the object 'my_plot'

my_plot <- ggplot(average_datarequests, aes(x = reorder(locations, -freq), y = freq)) +
  geom_bar(stat = "identity", fill = custom_color) +
  labs(x = "MIRACUM DIC Sites (Ordered by Frequency)", 
       y = "Average Frequency of Data Use Projects per Quarter") +
  theme_minimal() +
  theme(axis.title.x = element_text(size = 10, face = "bold", family = "Arial"),
        axis.title.y = element_text(size = 10, face = "bold", family = "Arial"),
        panel.grid.major = element_blank(),
        panel.grid.minor = element_blank(),
        panel.border = element_blank(),
        axis.line = element_line(colour = "black"),
        text = element_text(family = "Arial"),
        plot.title = element_text(hjust = 0.5, size = 16, face = "bold"),
        axis.text.x = element_text(angle = 45, hjust = 1, size = 10)) +
  scale_y_continuous(expand = expansion(add = c(0.05, 0.2))) +
  scale_fill_manual(values = custom_color) +
  coord_flip()

# Export the plot as a JPG file
ggsave("my_plot.jpg", plot = my_plot, width = 8, height = 6, dpi = 900)

# Display the resulting plot
my_plot
```


LS0tCnRpdGxlOiAiR3JhcGhpY2FsIEFuYWx5c2lzIGZyb20gc3VydmV5IGRhdGEiCm91dHB1dDogaHRtbF9ub3RlYm9vawotLS0KCgoKYGBge3IgbG9hZGluZyB0aGUgcmVxdWlyZWQgcGFja2FnZXN9CmxpYnJhcnkocmVhZHIpCmxpYnJhcnkodGlkeXZlcnNlKQpsaWJyYXJ5KGhhdmVuKQpsaWJyYXJ5KGdncGxvdDIpCmBgYAoKYGBge3IgSW1wb3J0IGF2ZXJhZ2UgZGF0YX0KCmF2ZXJhZ2VfZGF0YXJlcXVlc3RzIDwtIHJlYWRfY3N2KCJhdmVyYWdlX2RhdGFyZXF1ZXN0cy5jc3YiKQpWaWV3KGF2ZXJhZ2VfZGF0YXJlcXVlc3RzKQoKCgphdmVyYWdlX2RhdGFyZXF1ZXN0cyA8LSB0cmFuc2Zvcm0oYXZlcmFnZV9kYXRhcmVxdWVzdHMsCiAgICAgICAgICAgICAgICAgICAgICAgICAgICAgICAgICB2YXJpYWJsZT1yZW9yZGVyKGxvY2F0aW9ucywgLWZyZXEpICkgCmBgYAoKCmBgYHtyIFBsb3QgZXhlY3V0aW9ufQoKCiMgU2V0IHRoZSBjdXN0b20gY29sb3IKY3VzdG9tX2NvbG9yIDwtICIjRTA2NkZGIgoKIyBQZXJmb3JtIHRoZSBnZ3Bsb3QgdmlzdWFsaXphdGlvbiAKIyBTYXZpbmcgdGhlIG91dGNvbWUgaW50byB0aGUgb2JqZWN0ICdteV9wbG90JwoKbXlfcGxvdCA8LSBnZ3Bsb3QoYXZlcmFnZV9kYXRhcmVxdWVzdHMsIGFlcyh4ID0gcmVvcmRlcihsb2NhdGlvbnMsIC1mcmVxKSwgeSA9IGZyZXEpKSArCiAgZ2VvbV9iYXIoc3RhdCA9ICJpZGVudGl0eSIsIGZpbGwgPSBjdXN0b21fY29sb3IpICsKICBsYWJzKHggPSAiTUlSQUNVTSBESUMgU2l0ZXMgKE9yZGVyZWQgYnkgRnJlcXVlbmN5KSIsIAogICAgICAgeSA9ICJBdmVyYWdlIEZyZXF1ZW5jeSBvZiBEYXRhIFVzZSBQcm9qZWN0cyBwZXIgUXVhcnRlciIpICsKICB0aGVtZV9taW5pbWFsKCkgKwogIHRoZW1lKGF4aXMudGl0bGUueCA9IGVsZW1lbnRfdGV4dChzaXplID0gMTAsIGZhY2UgPSAiYm9sZCIsIGZhbWlseSA9ICJBcmlhbCIpLAogICAgICAgIGF4aXMudGl0bGUueSA9IGVsZW1lbnRfdGV4dChzaXplID0gMTAsIGZhY2UgPSAiYm9sZCIsIGZhbWlseSA9ICJBcmlhbCIpLAogICAgICAgIHBhbmVsLmdyaWQubWFqb3IgPSBlbGVtZW50X2JsYW5rKCksCiAgICAgICAgcGFuZWwuZ3JpZC5taW5vciA9IGVsZW1lbnRfYmxhbmsoKSwKICAgICAgICBwYW5lbC5ib3JkZXIgPSBlbGVtZW50X2JsYW5rKCksCiAgICAgICAgYXhpcy5saW5lID0gZWxlbWVudF9saW5lKGNvbG91ciA9ICJibGFjayIpLAogICAgICAgIHRleHQgPSBlbGVtZW50X3RleHQoZmFtaWx5ID0gIkFyaWFsIiksCiAgICAgICAgcGxvdC50aXRsZSA9IGVsZW1lbnRfdGV4dChoanVzdCA9IDAuNSwgc2l6ZSA9IDE2LCBmYWNlID0gImJvbGQiKSwKICAgICAgICBheGlzLnRleHQueCA9IGVsZW1lbnRfdGV4dChhbmdsZSA9IDQ1LCBoanVzdCA9IDEsIHNpemUgPSAxMCkpICsKICBzY2FsZV95X2NvbnRpbnVvdXMoZXhwYW5kID0gZXhwYW5zaW9uKGFkZCA9IGMoMC4wNSwgMC4yKSkpICsKICBzY2FsZV9maWxsX21hbnVhbCh2YWx1ZXMgPSBjdXN0b21fY29sb3IpICsKICBjb29yZF9mbGlwKCkKCiMgRXhwb3J0IHRoZSBwbG90IGFzIGEgSlBHIGZpbGUKZ2dzYXZlKCJteV9wbG90LmpwZyIsIHBsb3QgPSBteV9wbG90LCB3aWR0aCA9IDgsIGhlaWdodCA9IDYsIGRwaSA9IDkwMCkKCiMgRGlzcGxheSB0aGUgcmVzdWx0aW5nIHBsb3QKbXlfcGxvdApgYGAKCgoKCgoK
