# Supplementary figures and images for: Evaluating and Enhancing the Fitness-for-Purpose of Electronic Health Record Data: Qualitative Study on Current Practices and Pathway to an Automated Approach Within the Medical Informatics for Research and Care in University Medicine Consortium
Source: JMIR Med Inform. 2024 Aug 19;12:e57153. doi: 10.2196/57153 (PMC11369535; doi:10.2196/57153)

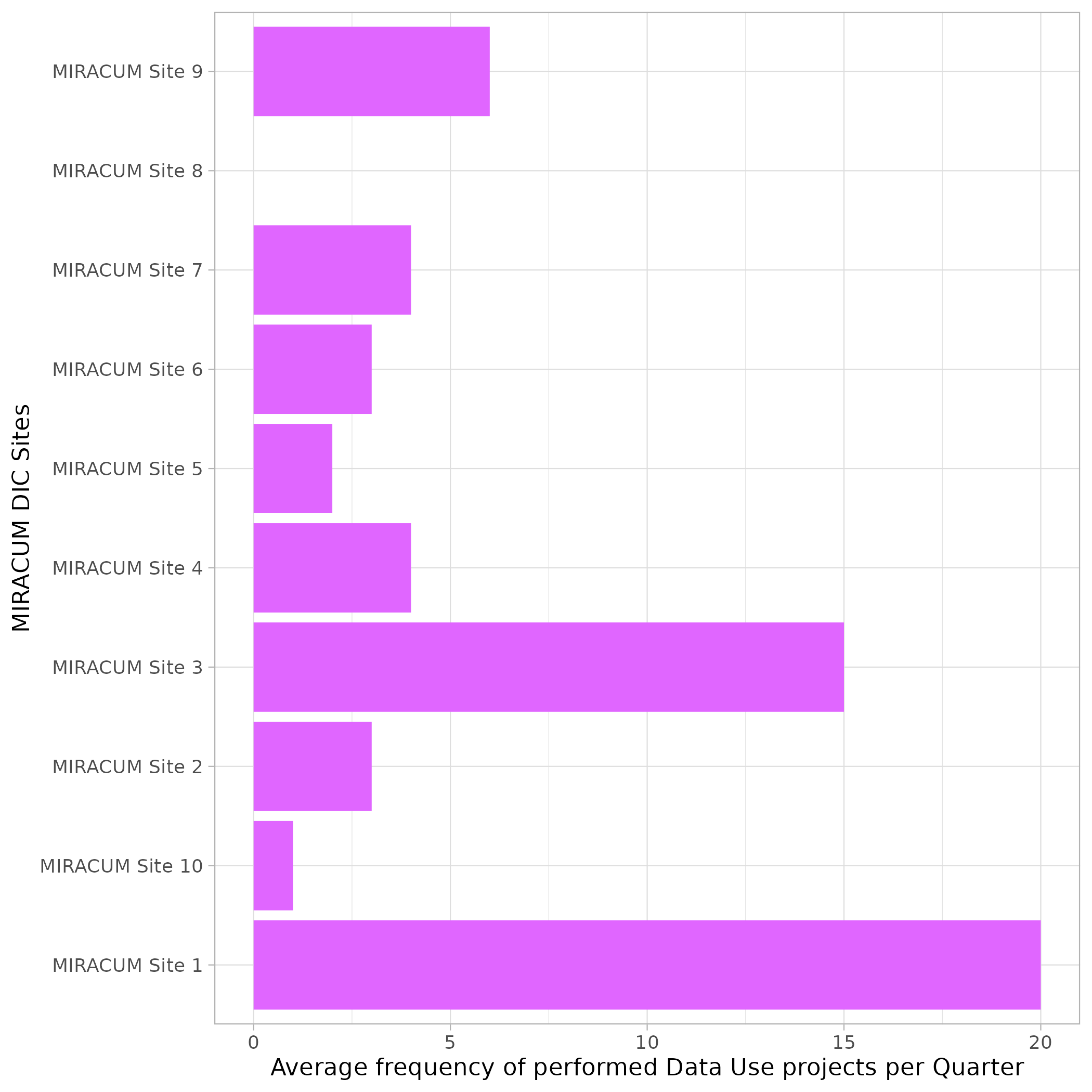

Supplement: Multimedia Appendix 4 [file medinform_v12i1e57153_app4.zip › analysis/analysis]

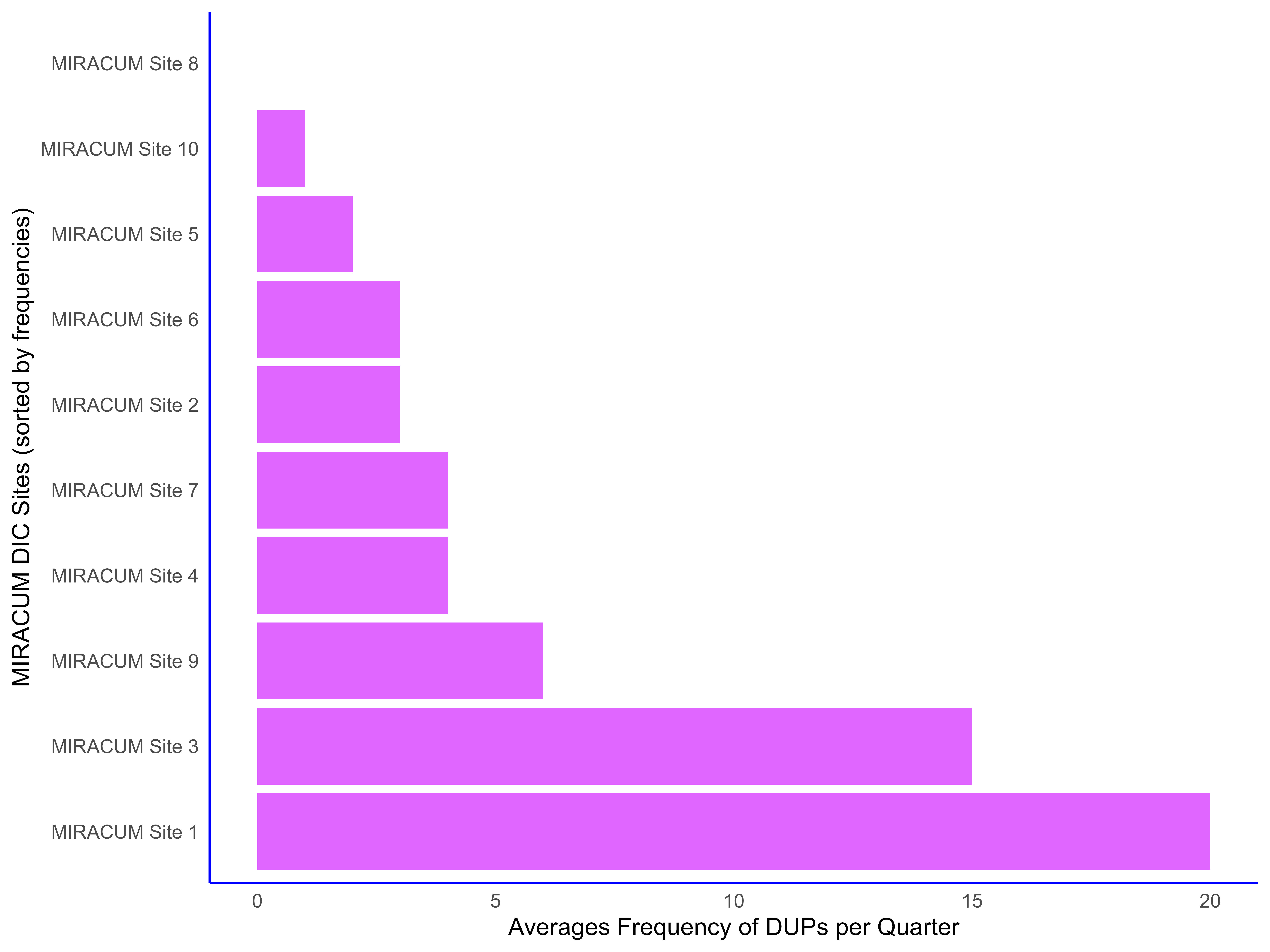

Supplement: Multimedia Appendix 4 [file medinform_v12i1e57153_app4.zip › analysis/freq_plot.jpg]
